# Protecting patient privacy when sharing patient-level data from clinical trials

**DOI:** 10.1186/s12874-016-0169-4

**Published:** 2016-07-08

**Authors:** Katherine Tucker, Janice Branson, Maria Dilleen, Sally Hollis, Paul Loughlin, Mark J. Nixon, Zoë Williams

**Affiliations:** Roche Products Ltd, 6 Falcon Way, Shire Park, Welwyn Garden City, AL7 1TW UK; Novartis Pharma AG, Basel, Switzerland; Pfizer Ltd, Walton Oaks, Dorking Road, Walton-on-the-Hill, Tadworth, Surrey, UK; AstraZeneca, Alderley Park, Cheshire, Macclesfield SK10 4TG UK; Centre for Biostatistics, Institute of Population Health, University of Manchester, Manchester Academic Health Science Centre, Oxford Road, Manchester, M13 9PL UK; Chilli Consultancy Ltd, Aldwych House, Winchester Street, Andover, Hampshire SP10 2EA UK; LEO Pharma, Horizon, Honey Lane, Hurley, SL6 6RJ UK

**Keywords:** Clinical trial, Data sharing, Transparency, De-identification, Anonymisation, Pharmaceutical research

## Abstract

**Background:**

Greater transparency and, in particular, sharing of patient-level data for further scientific research is an increasingly important topic for the pharmaceutical industry and other organisations who sponsor and conduct clinical trials as well as generally in the interests of patients participating in studies. A concern remains, however, over how to appropriately prepare and share clinical trial data with third party researchers, whilst maintaining patient confidentiality. Clinical trial datasets contain very detailed information on each participant. Risk to patient privacy can be mitigated by data reduction techniques. However, retention of data utility is important in order to allow meaningful scientific research. In addition, for clinical trial data, an excessive application of such techniques may pose a public health risk if misleading results are produced. After considering existing guidance, this article makes recommendations with the aim of promoting an approach that balances data utility and privacy risk and is applicable across clinical trial data holders.

**Discussion:**

Our key recommendations are as follows:

1. Data anonymisation/de-identification: Data holders are responsible for generating de-identified datasets which are intended to offer increased protection for patient privacy through masking or generalisation of direct and some indirect identifiers.
2. Controlled access to data, including use of a data sharing agreement: A legally binding data sharing agreement should be in place, including agreements not to download or further share data and not to attempt to seek to identify patients. Appropriate levels of security should be used for transferring data or providing access; one solution is use of a secure ‘locked box’ system which provides additional safeguards.

**Summary:**

This article provides recommendations on best practices to de-identify/anonymise clinical trial data for sharing with third-party researchers, as well as controlled access to data and data sharing agreements. The recommendations are applicable to all clinical trial data holders. Further work will be needed to identify and evaluate competing possibilities as regulations, attitudes to risk and technologies evolve.

## Background

### Introduction

This article is one of a series of articles developed by the EFSPI (European Federation of Statisticians in the Pharmaceutical Industry) [1] and PSI (Statisticians in the Pharmaceutical Industry) [2] Data Sharing Working Group. The working group consists of medical research statisticians from both pharmaceutical industry and academia with the intention of providing knowledge and insights regarding the practical challenges and opportunities of accessing clinical trial data for re-analysis or secondary scientific research purposes.

The intended audience for this article comprises of any holder of patient-level data generated from clinical trials (referred to as ‘data holder’), who wishes to share data for the purpose of secondary scientific research. It will also be useful background information for academic researchers who aim to access patient level data from clinical trials, in understanding what steps may have been applied to the data in order to protect patient privacy.

Greater transparency and, in particular, sharing of patient-level data for further research is an increasingly important topic for the pharmaceutical industry and other organisations who sponsor and conduct clinical trials (government agencies, academia, charities etc.). Drivers of these changes have come from several sources - for example, the scientific community/academia, e.g. Alltrials [3], BMJ (British Medical Journal) Open Data Campaign [4]; regulators, e.g. EMA (European Medicines Agency) policy 0070 [5] and the pharmaceutical industry e.g. PhRMA/EFPIA principles (Pharmaceutical Research and Manufacturers of America, European Federation of Pharmaceutical Industries and Associations) [6].

This paradigm shift aims to maximise the value of patient-level data from clinical trials for the benefit of future patients and society, by sharing clinical trial data with researchers for secondary research. At the same time it is also essential that this is balanced with the risk to the privacy and identity of individual patients. Thus a process step to protect patient data privacy is an essential prerequisite for data sharing in order to adequately safeguard the privacy of patients participating in clinical trials, whilst making data available for further research.

The article briefly considers existing legislation, guidance and common practices and then recommends best practices relevant to protecting patient privacy, when sharing clinical trial data. Our recommendations aim for an approach that balances data utility and privacy risk and is applicable to any holder of clinical trial data.

### Scope and assumptions

The following topics related to protecting patient privacy, when sharing patient-level data from clinical trials, are in-scope for this article:

- best practices for data anonymisation/de-identification
- the role of controlled access (e.g. via a secure ‘locked box’ system)
- legally binding data sharing agreements (DSAs)

It is important to recognise that the level of data de-identification required will relate to the level of security and safeguards in the method of sharing. For example, making data available on the internet, with no legal agreements in place, greatly increases the number of people with access to the data. Those accessing data may have a variety of motives for doing so, potentially including attempting to reveal patient identities, with no legal agreement to prevent this or control on access. Therefore, in this scenario, data must be prepared such that the risk of an ‘attacker’ being able to reveal patient identities is minimal (i.e. high levels of data anonymisation/de-identification). We do not consider this scenario further within this article.

The recommendations in this article assume the data are being shared in the context of genuine scientific research, based on agreed research objectives, with some minimum level of legal, data security and access safeguards being employed. In this scenario, the overall risk of a researcher being motivated to reveal patient identities is expected to be very low, and potentially any breach of privacy would have serious professional consequences. Therefore data can be prepared in such a way that greater utility is retained in order to enhance the integrity of resulting analyses and interpretation. However, the data holder is ultimately responsible for ethical and legal obligations and assessing risk of patient re-identification (based on level of security, safeguards, etc.).

The following are out of scope for this article, but will also be important considerations for data holders when sharing clinical trial data:

- broader aspects related to patient privacy when sharing clinical trial data, including informed consent, the criteria determining which studies are available for sharing, request tools and processes, statistical analysis software or tools provided, and legal aspects of DSAs. The PhRMA/EFPIA ‘Principles for Responsible Clinical Trial Sharing’ [6] and the Institute of Medicine report ‘Sharing Clinical Trial Data: Maximizing Benefits, Minimizing Risk’ [7] address several of these aspects. The companion EFSPI/PSI Working Group article Data Sharing: Accessing and Working with Pharmaceutical Clinical Trial Patient Level Datasets – a Primer for Academic Researchers (EFSPI/PSI Working Group on Data Sharing:Accessing and Working with Pharmaceutical Clinical Trial Patient Level Datasets – a Primer for Academic Researchers. In submission), provides an overview of topics such as finding information, writing a research proposal, the review process, how data are shared and expectations of the data holder.
- appropriate standards for security when sharing data.
- redaction and sharing of documents e.g. clinical study reports (CSRs) including consideration of ‘commercially confidential information’ which is discussed in more detail in TransCelerate [8] and EMA guidance [5, 9].

### Terminology and definitions

For the purposes of this article, we have used the following definitions:

#### Patient-level data

EMA policy 0070 [5] defines Individual Patient Data as “*the individual data separately recorded for each participant in a clinical study*”; for the purposes of this article we call this ‘patient-level data’. Examples of patient-level data collected in clinical trials are patient identifier, site identifier, date of birth, gender, race, efficacy outcomes, laboratory test results, etc.

#### Anonymised/de-identified data

Guidance on implementation of the EMA policy 0070 [9] defines anonymisation as “the process of rendering data into a form which does not identify individuals and where identification is not likely to take place”, and anonymised/de-identified data as “data in a form that does not identify individuals and where identification through its combination with other data is not likely to take place”.

The Health Insurance Portability and Accountability Act (HIPAA) [10] defines “*de-identified protected health information’ as ‘Health information that does not identify an individual….there is no reasonable basis to believe that the information can be used to identify an individual…*.”.

Note that terms ‘de-identification’ and ‘anonymisation’ are often used interchangeably in different contexts in the literature.

#### Raw and derived data

Raw data means patient-level data that have been directly collected during a clinical trial or study (e.g. weight and height of a patient). Derived data are data that is obtained from raw data and which have undergone a derivation or calculation (e.g. body mass index is derived from the weight and height of a patient). Patient-level data to be shared can include raw and/or derived data.

#### Data holder

Entity which holds the clinical trial data and has the ability and authority to share with third party researchers.

### Existing legislation, guidance and common practices

This section briefly outlines existing legislation, guidance and common practices.

There are various elements of EU (European Union) legislation and EMA (European Medicines Agency) policy which relate to data transparency [5, 11–13]. EU data privacy legislation [12, 13] states that *“The principles of (data) protection should not apply to data rendered anonymous in such a way that the data subject is no longer identifiable.”* In December 2015, the EU governmental institutions reached agreement on the text of the new General Data Protection Regulation that will replace the existing Directive [14]. This new Regulation was adopted by the EU Parliament and Council in May 2016 and will be become applicable in 2018. The precise impact of this new Regulation on matters discussed in this paper is still not totally clear but will emerge during the two year transition phase. Whilst EMA focus is currently on greater transparency of documents, EMA policy 0070 [5] does include potential future provision for sharing of patient-level data. It includes reference to EU article 29 WP216 [15], which is critiqued by Khaled El Aman [16], and the EMA’s guidance on implementation [9] addresses anonymisation of personal data of trial participants which will be relevant to the sharing of patient-level data.

US Policy 45 CFR part 46, also known as the ‘Common Rule’ [17], requires de-identification of data prior to release for further research. US ‘HIPAA’ privacy rule [10] outlines two approaches commonly applied:’Expert Determination’ and ‘Safe Harbor’. The’Expert Determination’ approach requires a statistical expert to apply statistical and scientific principles in order to render data not individually identifiable or such that the risk of re-identification is very small. The ‘Safe Harbor’ approach requires removal of eighteen direct identifiers which could be used to identify the individual or the individual’s relatives, employers, or household members, many of which are not routinely collected in clinical trials and applies to US populations. The eighteen ‘Safe Harbor’ direct identifiers are outlined in Table 1. Building on the principles outlined in HIPAA, Hrynaszkiewicz [18] considers both direct and indirect identifiers, with a focus on publication of data for unrestricted access.

**Table 1:** HIPAA eighteen direct identifiers

A. Name
B. Geographic subdivisions smaller than a state. The initial three digits of a ZIP code can be retained if certain criteria are met.
C. With the exception of year, all elements of dates directly related to an individual (such as birth date, admission date, discharge date, date of death). For ages over 89 and elements of dates (including year) indicating such an age, ages and elements may be aggregated into a single category of age 90 or older.
D. Telephone numbers,
E. Fax numbers
F. Email addresses
G. Social security numbers
H. Medical record numbers
I. Health plan beneficiary numbers
J. Account numbers
K. Certificate/licence numbers
L. Vehicle identifiers and serial numbers, including license plate numbers
M. Device identifiers and serial numbers
N. Web Universal Resource Locators (URLs)
O. Internet Protocol (IP) addresses
P. Biometric identifiers, including finger and voice prints
Q. Full-face photographs and any comparable images
R. Any other unique identifying number, characteristic, or code, except as permitted by paragraph (c) of HIPAA Safe Harbor section; and
S. The covered entity does not have actual knowledge that the information could be used alone or in combination with other information to identify an individual who is a subject of the information

The Institute of Medicine report ‘Sharing Clinical Trial Data: Maximizing Benefits, Minimizing Risk’ [7] includes an appendix ‘Concepts and Methods for De-identifying Clinical Trial Data’, which focuses on quantifying risk of re-identification. Mello [19] and the IPPC (International Pharmaceutical Privacy Consortium) report [20] also consider the de-identification of clinical trial data, whilst NIH (National Institute of Health) requires its investigators to deliver data sets prepared in order to reduce the risk of re-identification [21].

Various approaches to transforming data to preserve privacy of the subjects have been proposed across a broad range of data sharing situations. Aggarwal and Yu [22] and the Institute of Medicine [7] provide examples of approaches to achieve anonymity of personal health information, including the following:

- Transformation of data sets using data reduction techniques such as generalisation of the data by grouping of values into categories, and suppression/masking of data where specific values or whole records are removed from the dataset. Data perturbation techniques can also be applied, whereby random noise is added to the true values.
- Diversity/closeness models have been developed with the aim of transforming data to ensure that specific individuals cannot be identified within public databases [23]. These provide a guarantee of a pre-specified level of anonymity based on non-uniqueness of records within the transformed data.
- Provision of aggregate results, such as area level census data.
- Provision of the results of data analysis such as data mining through applications which hide certain data attributes rather than direct access to data.

There are a variety of existing models for requesting access to pharmaceutical clinical trial data; some examples include ClinicalStudyDataRequest.com [24], ‘YODA’ (Yale University Open Data Access) [25], Pfizer [26]. On these websites, companies outline their procedures for de-identifiying/anonymising clinical trial data (see example outlining GSK (GlaxoSmithKline) principles [27], also adopted by some other data holders with some variations). These examples are broadly representative of the steps implemented by many pharmaceutical companies. Guidance aimed at UK publically funded clinical trials units has been published, funded by the UK MRC Network of Hubs for Trials Methodology Research [28]. ‘The 5 Safes’ [29] outlined by the UK Data Service, holistically considers ‘Safe People’, ‘Safe Projects’, ‘Safe Settings’, ‘Safe Outputs’ and ‘Safe Data’, when sharing sensitive data for research.

TransCelerate [30] and PhUSE (Pharmaceutical Users Software Exchange) [31] are pharmaceutical industry bodies with working groups who have recently developed models or standards around data de-identification. Whilst TransCelerate is focusing on general principles, PhUSE focuses on specific implementation rules in CDISC SDTM 3.2 (Clinical Data Interchange Standards Consortium, Study Data Tabulation Model) and provides guidance on determining which data elements may be direct or indirect identifiers. Whilst these guidances focus primarily on pharmaceutical clinical trial data, the general principles could be more generally applied.

## Discussion

This section outlines key recommendations in two areas: anonymisation/de-identification of patient-level data for third-party research and controlled access to data, including use of a DSA.

### Data anonymisation/de-identification

To create these recommendations for best practices for anonymisation/de-identification of patient-level clinical trial data, we have considered:

- relevant legislation, guidance and common practices,
- context in which data holders share data (e.g. approved research proposals, legal and data security controls),
- practical considerations related to the ability to efficiently and effectively prepare and deliver large volumes of data requests in a semi-automated fashion,
- ability to align/standardise processes across data holders,
- cost and resource implications,
- how to retain data utility.

Patient confidentiality can never be 100 % guaranteed, especially as the general availability of data in the public domain increases over time, including social media. If these external data were to be combined with the de-identified clinical trial data provided, it may increase the risk of patient re-identification. Data holders take the protection of patient privacy seriously. Whilst no data holder can completely rule out illegitimate attempts to re-identify data, they can employ a number of strategies to minimise the risk and this article highlights those we consider as best practice.

This guidance aims to provide pragmatic and effective recommendations in order to minimise breaches of patient confidentiality (e.g. revealing of the true identity of a patient), whilst maximising data utility (e.g. ability of a researcher to conduct meaningful analyses). Applying high levels of data de-identification may result in data that is not useful for answering scientific questions, or results in misleading interpretation. Conversely, provision of useful data with lower levels of de-identification, may raise the risks around patient confidentiality. This article attempts to strike a pragmatic balance between the two.

In making our recommendations, we assume the following:

- The process for receiving, reviewing and approving any required research proposal has been completed. Data will only be shared in relation to ‘approved’ research proposals with named researchers, only whom will have access to the data.
- Data will be shared in a secure and controlled manner under a DSA with legal safeguards on misuse of data (see later section).
- Both raw and analysis-ready datasets may be provided to researchers and the recommendations apply to both types of data. However, genetic data are out of scope for this article as we consider that the potential for sensitive information to be released (e.g. previously unknown genetic abnormalities which may have serious implications on that person’s future health) and the risk of patient re- identification to be too high (as the set of genetic results will always be unique to each patient).

Clinical trials often involve collection of data from patients across geographical boundaries. Researchers will also reside across the globe. As such, data anonymisation/de-identification rules need to be applicable to data collected in any country and also potentially shared with researchers residing across the world.

The HIPAA Privacy Rule [10] outlines two approaches to de-identification of data,’Expert Determination’ and ‘Safe Harbor’. It is recognised that there are particular difficulties in applying’Expert Determination’ algorithms to high-dimensional data. Eze and Peyton [32] note that most diversity/closeness models fail with high-dimensional health data because the non-uniqueness that is required “results in excessive information loss, virtually wiping off the analytical utility of the dataset”. This is a particular issue for clinical trial data due to the nature of the high-dimensionality, and because some types of data are critical for data utility but not amenable to a non-uniqueness approach. Compared to routinely collected health data, the ratio of the number of data fields to the number of subjects will be considerably higher creating a more extreme challenge. Some critical information such as rare adverse events have an inherently high level of uniqueness, and we consider that the use diversity/closeness models would generalise the data unacceptably.

Thus our recommendation is to utilise a de-identification approach in line with HIPAA ‘Safe Harbor’, with further generalisation and masking of some indirect identifiers. Whilst initially written with the US population in mind, this approach lends itself to some level of automation and consistent approach across studies. This can potentially enable quicker processing and delivery of large volumes of studies and datasets and therefore provides a good starting point for our recommendations. It also better enables subsequent pooling/meta- analyses as all de-identified/anonymised studies can be handled uniformly, whereas expert determination may result in differing levels of de-identification/anonymisation, for example based on differing patient populations.

Whilst our aim in this article is to recommend steps which can be automated to some extent, it is important that data holders do not consider the process as purely a ‘push button’ exercise. Preparing data for sharing will always involve some element of manual review in order to identify and remove unexpected identifiers in the data, particularly for legacy data which maybe have less standardisation. In addition, data holders should review their data anonymisation/de-identification steps regularly to ensure they remain in line with current thinking and guidance.

The specific quantification of risk (of patient re-identification), the acceptable risk threshold and the methods employed to do so are the responsibility of the data holder.

### Recommended best practices

We consider the following as best practices for anonymisation/de-identification, however the specific steps taken will depend on the individual circumstances of the request, how the data will be shared and other trial-related information.

- **Direct identifiers:** Any of the HIPAA direct identifiers collected in clinical trial data should be removed. Dates are handled as described below. The eighteen direct identifiers are outlined in Table 1.
- **Patient identifiers:** In clinical trials, each patient is given a unique number or code (patient code number/identifier) and these are classed as direct identifiers. They are generally used across datasets. To maintain the link across records between datasets, patient identifier codes should be replaced with new randomly generated numbers/identifiers consistently across datasets and extension studies (where applicable). The new patient identifier should not depend at all on information contained in the corresponding records. This ensures there is no identifier present in the data which directly links to the original patient-level data and patient notes which, for example, would be present at the clinical trial site, together with other direct identifiers such as name and address.
- **Code key:** The data holder should ensure that the ‘code key’ that was used to generate the new code number from the original is securely stored, or destroyed. As noted by the Institute of Medicine, the data holder will, by definition, be able to re-identify the data as they retain the original trial data. Thus the minimum standards of security if the ‘code key’ is retained are those applied to the original data such as storage in a secure location with access by only limited and authorised personnel.
- **Dates:** Date of birth is information that might lead to identification of a specific patient and therefore it should be replaced with age. Other patient related dates, including date of death, should be replaced by study day relative to a reference date or by offset dates (the offset approach is described further in TransCelerate report ‘Data De-identification and Anonymization of Individual Patient Data in Clinical Studies – A Model Approach’ [30] and PhUSE De-identification Standard for SDTM 3.2 [31]). The PhUSE standard also contains commentary comparing study day versus offset methods, and describes some of the issues with offset dates. EMA guidance on implementation of their policy on publishing clinical trial data suggests that the offset dates should be within the range of dates occurring during the trial, which may require complex algorithms in some cases such as variable duration of follow-up. Currently, the use of one method over another is based on data holder preference. The PhUSE standard states that it is preferable, from a data utility perspective, to keep both actual dates with offsets applied and relative study day. Further work is required to assess the implications of different approaches to generating offsets and their implications for data utility and the risk of re-identification. Study day retains much of the scientific value in the date information, while avoiding the uncertain risk that may arise from the use of offsets. Since removal of information relating to actual dates may significantly reduce data utility in seasonal type diseases such as influenza or hayfever, alternative approaches may need to be considered in these scenarios e.g. the data holder may derive and retain month or season information.
- **Indirect identifiers:** Specific recommendations for indirect identifiers can be found in Table 2. Potential indirect identifiers which are important for data utility may be retained and could be recoded/grouped, otherwise they should be removed.

**Table 2:** Specific recommendations for indirect identifiers

Site Code Number/Investigator Identifier
• In clinical trial data, place of treatment is usually collected as a site code number/investigator identifier. These site codes should be re-coded to a new random site code (similar to patient code number/identifier). Sites which include few patients may be aggregated to a single site code number/identifier. Countries which include few patients could also be pooled.
Demographics and anthropometry measures
• Date of birth is a direct identifier and should be should be replaced with age. As a general rule, ages above 89 should be set to a category ‘ > 89’; however depending on the disease and the population under consideration, further grouping of age categories should be considered. Consideration should also be given to recoding/grouping other ages at the lower or upper limits. Another consideration, assuming this does not impact data utility adversely, is to group ages (for example into five year age categories). All other patient-related dates including date of death should be removed and replaced either with a derived study day relative to a baseline or reference date or offset by some random interval. • Gender can be kept and it is recommended that race is mapped into categories (e.g. FDA recommendations: American Indian or Alaska Native, Asian, Black or African American, Native Hawaiian or Other Pacific Islander, and White). Ethnicity is usually removed. • Anthropometry measures (e.g. weight, height) should be kept in de-identified datasets as they are frequently key variables for dosing (e.g. mg/kg) or present in the calculations of body surface area (BSA) or body mass index (BMI) or used as covariates in data analyses. Consider grouping variables.
Verbatim text
• Verbatim (free) text may include information that identifies a patient e.g. names, dates or other personal information. Examples of variables containing verbatim text are adverse events, medications, medical history and general comments. Preferably, variables containing free text are either removed from the dataset or set to blank. Alternatively, the data could be reviewed to assess the risk of patient identification, especially if the data add scientific value to the dataset, and any identifiers at the observational level removed. • Where a variable has been coded according to a standard dictionary (e.g. adverse events to MedDRA, medications to WHO ATC), the dictionary term(s) should be retained in the dataset and the verbatim text dropped.
Small populations, low frequency and rare events, rare diseases, sensitive data
• Hrynaszkiewicz recommends that for indirect identifiers with small denominators (population size of <100) or very small numerators (event counts of <3), may present a risk if present in combination with other indirect identifiers. However, to exclude such data in all cases may limit the ability of a researcher to perform meaningful analyses, particularly in the case of small numerators for adverse event reporting which may result in the removal of rare events of interest. • Studies involving rare diseases or including sensitive data should be reviewed on a case by case basis and assessed as to whether sufficient steps can be taken to adequately maintain patient confidentiality.
Other
• Potential indirect identifiers which are important for data utility may be retained and could be recoded/grouped, otherwise they should be removed.

### Process, quality control and documentation

It is important the data holders define a process for data anonymisation/de-identification, execute that process and then have a separate quality control step to review and document correct implementation of the process. During this process of preparation of the data, programmers will need to refer to both original and new datasets and once completed, the ‘code key’ linking both sets of data is deleted or secured. This step may also include assessment of risk of patient re-identification.

Ideally, we recommend that to reduce the risk of re-identification, data holders only provide a subset of the data as required to fulfil the research proposal. However, for efficiency reasons, data holders may wish to provide all datasets for a study. Whilst providing the full set of data may increase risk of re-identification, it also ensures that the researcher can draw on any data that are relevant to their interpretations, even if they had not anticipated the specific need for that data. Even if a subset of the data are shared, it may be more efficient to de-identify/anonymise an entire study at once, so it is ready for future requests, particularly in circumstances where the data holder chooses to destroy the linking code key.

Supporting documentation such as redacted versions of the Clinical Study Report (CSR), protocol, statistical analysis plan, dataset specifications, annotated CRFs (case report form) and in certain cases statistical programs (or sections of ‘pseudo’ code which outlines key derivations or statistical models fitted) should be provided to researchers, where available, in order to help researchers navigate and understand the datasets.

In addition, documentation should be provided outlining steps taken to de-identify/anonymise the data and further details if only a subset of the data is provided. For example, a report might outline the variables removed or aggregated for each dataset with details of how variables had been altered (e.g. age replaced by age categories). This is important given that the original dataset specifications will not exactly match the newly de-identified/anonymised data provided. Documents e.g. the main text of CSRs should be redacted at least to the same extent as patient-level data (e.g. all patient identifiers to be removed) and patient level listings and tabulations should be completely removed. Further guidance on redaction of CSRs is available [8, 9].

### Controlled access to data, including use of a data sharing agreement (DSA)

This section outlines our recommendations for providing the researcher controlled access to clinical trial data, including use of DSAs. In order to protect patient privacy, access to data should be provided following these minimum recommendations:

1. A DSA (also sometimes known as data use agreement or DUA) should be signed by data holder and researchers, which includes sufficient limitations on what the recipients can and cannot do with the data. This should relate to a specific research proposal which has already been agreed and approved.
2. Only named (and appropriately qualified) researchers should be granted access to the data.
3. Appropriate levels of security should be used when transferring data or providing access to data.

Whilst a secure ‘locked box’ system (e.g. SAS Clinical Trial Data Transparency (CTDT) Multi Sponsor Environment (MSE)) [33] is not a necessity, it does provide additional safeguards to maintain patient privacy, as well as other features such as provision of analytical software and ability to simultaneously share data from multiple data holders with researchers. Features may include secure transfer of data and documents, ability to grant and revoke access to specific researchers (i.e. secure password protected login), ability to restrict downloading and cut and pasting of patient-level data to researchers’ personal computer, an audit trail accessible by the data holder etc. If a secure ‘locked box’ system is not utilised, then additional conditions to incorporate into the DSA, are outlined below.

A legally binding DSA should include at a minimum:

- Agreement to only use the data for the agreed purpose (based on an approved research proposal) and not to download, transfer or share the data for future use with anyone else
- Under no circumstances to attempt to seek to identify patients
- Individual passwords only to be used by assigned individuals to restrict access to data and in the case of secure transfer not to share with others
- If a secure system is not used, then additional conditions may be required such as:
  - ○ researchers must agree to assume responsibility for securely storing and managing access to data and documents;
  - ○ the DSA may be extended to name all individuals who will access the data (in secure systems, this may be manageable via system controls);
  - ○ researchers to prevent dissemination of datasets to individuals who are not identified in the DSA;
  - ○ appropriate management of data and documents once all research is complete (i.e. destruction/deletion from systems)
- In addition to concerns regarding patient privacy, consideration should be given to the process for handling potential new safety signals if identified (e.g. to inform the data holder immediately), researchers providing the data holder with a copy of the subsequent publication (for information and/or review) and citation, agreements regarding any inventions arising from the research and acknowledgements referring to provision of the data by the data holder.

### Future considerations

One overarching aim for the future is to maximise the ability of researchers to access consistently structured and prepared data from multiple sources, including pharmaceutical and non-commercial trial data (e.g. academia, government bodies, and charities). Therefore, EFSPI/PSI fully supports efforts to align processes, tools and systems across data holders with an aim for eventual alignment around a central repository, thereby allowing further sharing of experiences and costs and improving the experience and accessibility for researchers.

A topic which will continue to evolve is the extent to which data holders quantify risk of patient re-identification through the use of data sources and data linkage techniques that are available now, or those that may become available in the future. For data that are being shared publically, this could include the risk of a deliberate attack using data from public records and social media could leading re-identification. However, as discussed above, this scenario is considerably less likely when data sharing through controlled access by qualified researchers for an agreed purpose. As methods develop further, it may be possible for risk quantification to move from a qualitative to a quantitative assessment for clinical trial data de-identification. Indeed, clinical trials should be planned and executed with eventual data sharing aims built in, in line with the Institute of Medicine [7] recommendation that ‘*Stakeholders in clinical trials should foster a culture in which data sharing is the expected norm*’, and the International Committee of Medical Journal Editors (ICMJE) proposal to require authors to share with others de-identified individual-patient data underlying the results presented in articles reporting clinical trials [34].

Regulatory guidance and legalisation is an evolving area which may eventually mandate minimum steps for data de-identification and anonymisation to facilitate patient-level data sharing. EFSPI/PSI are keen to participate and contribute to any future consultation with EMA on the provision of patient-level data.

It is recognised that the resources, costs and effort required to make patient-level data available for third party research may be considerable, particularly during the early days of implementation of processes and practices. The burden on smaller data holders will be proportionately greater than larger companies or groups with more resources at their disposal. However, as outlined in this article, regulations may eventually mandate sharing of patient-level data from pharmaceutical companies and so starting this debate and sharing recommendations now may allow data holders to be in a good position to be able to address future regulatory needs.

Increasing standardisation of data collection, tabulation and reporting, at least in the pharmaceutical industry, should consequently mean data de-identification and anonymisation steps can also be further automated, thereby eventually reducing costs in preparing data for sharing. An increasing number of contract research organisations are also offering services and tools to de-identify and anonymise data as well as perform quantitative risk assessments when required.

## Summary

This article outlines best practices for data anonymisation/de-identification and controlled access to data including the use of DSAs, in order to protect patient privacy when sharing clinical trial data with third-party researchers. The guidance is applicable to any holder of clinical trial data, with the aim of promoting an approach that balances data utility and privacy risk and is applicable across data holders.

In creating these recommendations, we considered existing legislation, guidance and common practices relevant to protecting patient privacy; the context in which data holders share data (e.g. approved research proposals, legal and data security controls); practical considerations related to the ability to efficiently and effectively prepare and deliver large volumes of data requests in a semi-automated fashion; ability to align/standardise processes across data holders; cost and resource implications, and finally how to maximise data utility and hence the integrity of resulting analyses and interpretation.

Our key recommendations are:

1. Data holders are responsible for generating de-identified and anonymised datasets which protect patient privacy. Direct and some indirect identifiers must be removed from datasets. This includes recoding patient identifier codes (by replacing the original code number with a new code number), removing free text verbatim terms, replacing date of birth with age (and possibly grouping) and replacing all patient related dates with study day or offset dates. Potential indirect identifiers which are important for data utility may be retained and could be recoded/grouped, otherwise they should be removed.
2. A legally binding data sharing agreement should be in place, including agreements not to download or further share data and not to attempt to seek to identify patients. Access to data should be transferred and provided in a secure manner (data holders should password protect data at a minimum); one solution is use of a secure ‘locked box’ system which provides additional safeguards.

We consider that these pragmatic recommendations strike a reasonable balance between maintaining patient privacy and retaining data utility, in order for the data to be as valuable as possible for further secondary research, the ultimate objective of data sharing. Further work will be needed to identify and evaluate competing possibilities as regulations, attitudes to risk, and technologies evolve.

## Abbreviations

BMJ, British medical journal; CCI, commercially confidential information; CDISC, clinical data interchange standards consortium; CFR, code of federal regulations; CRF, case report form; CSR, clinical study report; CTDT, clinical trial data transparency; DSA, data sharing agreement; EFPIA, European federation of pharmaceutical industries and associations; EFSPI, European federation of statisticians in the pharmaceutical industry; EMA, European medicines agency; EU, European union; GSK, glaxosmithkline; HIPAA, health insurance portability and accountability act; IPPC, international pharmaceutical privacy consortium; NIH, national institute of health; PhRMA, pharmaceutical research and manufacturers of America; PhUSE, pharmaceutical users software exchange; PSI, statisticians in the pharmaceutical industry; SDTM, study data tabulation model; SFTP, secure file transfer protocol; UK, United Kingdom; US, United States; YODA, Yale University Open Data Access
